# No Survival Benefit Associated With Antifungal Therapy for Respiratory *Candida* spp.: a Single-Center Retrospective Cohort Study

**DOI:** 10.1093/ofid/ofag550

**Published:** 2026-09-03

**Authors:** Jared Coe, Jaime Mogollon, Shilpa Vasishta, Christopher Vo, Kelsie Cowman, Phyu Thwe, Liise-anne Pirofski, Hyunah Yoon

**Affiliations:** Division of Infectious Diseases, Department of Medicine, Albert Einstein College of Medicine and Montefiore Medical Center, Bronx, New York, USA; Division of Infectious Diseases, Department of Medicine, Albert Einstein College of Medicine and Montefiore Medical Center, Bronx, New York, USA; Division of Infectious Diseases, Department of Medicine, Albert Einstein College of Medicine and Montefiore Medical Center, Bronx, New York, USA; Division of Infectious Diseases, Department of Medicine, Albert Einstein College of Medicine and Montefiore Medical Center, Bronx, New York, USA; Division of Infectious Diseases, Department of Medicine, Albert Einstein College of Medicine and Montefiore Medical Center, Bronx, New York, USA; Department of Pathology, Albert Einstein College of Medicine and Montefiore Medical Center, Bronx, New York, USA; Division of Infectious Diseases, Department of Medicine, Albert Einstein College of Medicine and Montefiore Medical Center, Bronx, New York, USA; Department of Microbiology and Immunology, Albert Einstein College of Medicine, Bronx, New York, USA; Division of Infectious Diseases, Department of Medicine, Albert Einstein College of Medicine and Montefiore Medical Center, Bronx, New York, USA

**Keywords:** antifungal therapy, *Candida* spp, immortal time bias, mortality, respiratory culture

## Abstract

**Background:**

*Candida* spp. are often recovered from respiratory cultures and usually interpreted as colonization. Whether species identity adds prognostic information or identifies patients more likely to benefit from antifungal therapy remains uncertain.

**Methods:**

We performed a single-center retrospective cohort study of hospitalized adults with respiratory *Candida* spp. from 2018 through 2023, excluding same-admission candidemia. Mortality was assessed through 30 and 90 days. Species and mortality associations were estimated with multivariable Cox models. Antifungal therapy was evaluated as a post-culture time-dependent exposure and in stabilized inverse-probability-weighted analyses.

**Results:**

Among 548 patients (median age, 66 years; 54.9% male; 33.4% non-Hispanic Black; 33.6% Hispanic), 78.3% required mechanical ventilation, 72.3% received vasopressors, and 30-day mortality was 27.0%. *C albicans* accounted for 478 isolates (87.2%), *C glabrata* for 18 (3.3%), and other non-albicans *Candida* spp. for 52 (9.5%). Compared with *C albicans*, *C glabrata* was associated with higher 30-day mortality (adjusted hazard ratio [aHR], 2.19; 95% confidence interval [CI], 1.05–4.56) and 90-day mortality (aHR, 2.18; 95% CI, 1.11–4.27). Antifungal therapy was not associated with lower 30-day mortality in time-dependent analysis (aHR, 1.25; 95% CI, 0.80–1.95) or weighted analysis (HR, 1.06; 95% CI, 0.64–1.75); 90-day analyses were also null.

**Conclusions:**

Respiratory *Candida* spp. identified severe illness. *C glabrata* identified a small high-mortality subgroup, but systemic antifungal therapy was not associated with improved survival, supporting caution against treating respiratory culture positivity alone.

## Teaser

We found that *Candida* spp. in respiratory cultures identified a critically ill population with 46% 90-day mortality, similar to candidemia, but systemic antifungal therapy was not associated with improved survival. These findings do not support systemic antifungal therapy based on respiratory culture positivity alone.

## Graphical Abstract

**Graphical Abstract:**
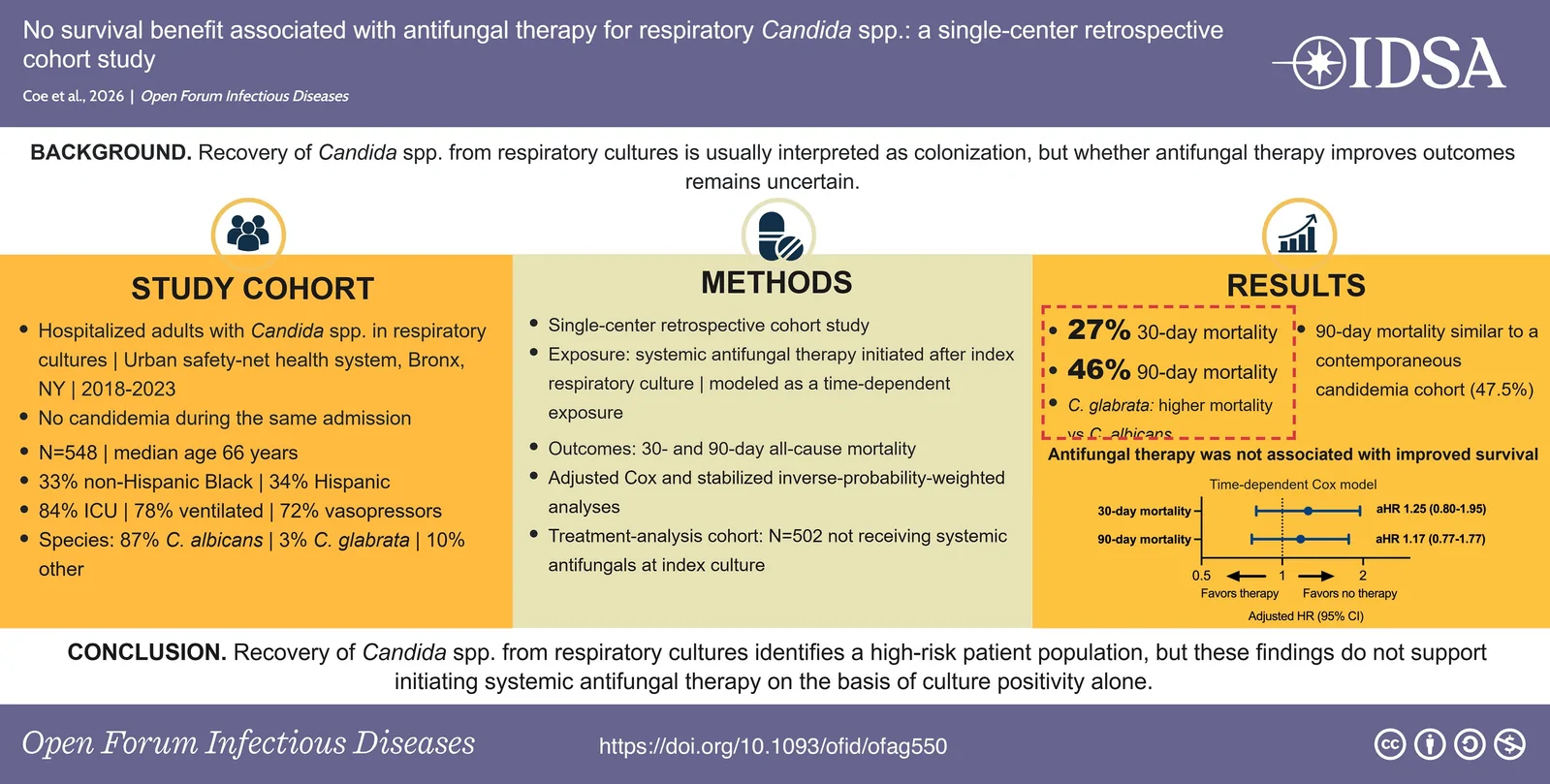
This graphical abstract is also available at Tidbit: https://tidbitapp.io/tidbits/no-survival-benefit-associated-with-antifungal-therapy-for-respiratory-candida-spp-a-single-center-retrospective-cohort-study-5447794e-fb91-455c-b790-f336d485b48d?utm_campaign=tidbitlinkshare&utm_source=ITP

Invasive candidiasis is a major healthcare-associated fungal infection; candidemia, its most common manifestation, carries a crude mortality of approximately one-third and approaching 40% in contemporary cohorts [1–4]. Recovery of *Candida* spp. from respiratory cultures is a different and more common bedside problem. *Candida* spp. are reported in up to half of intensive care unit (ICU) respiratory specimens in some series, yet guidelines discourage antifungal therapy for respiratory cultures alone because most represent colonization [5–10].

Still, the label of colonization may obscure clinically relevant host and microbial heterogeneity. Respiratory *Candida* spp. may reflect airway injury, aspiration, impaired clearance, antibiotic pressure, fungal burden, host vulnerability, or polymicrobial airway microbiology. Prior cohorts have associated respiratory *Candida* spp. with prolonged ventilation or hospitalization [11, 12], bacterial pneumonia or selection of antibiotic-resistant bacteria [13, 14], and a worse clinical course, although mortality associations have been inconsistent [11, 12]. Experimental data support biologic plausibility through *Candida*-bacterial biofilms [15, 16], enhanced bacterial virulence [16, 17], and impaired macrophage function [18]. Non-*albicans Candida* spp., particularly *C glabrata*, are increasingly important in invasive candidiasis because of reduced susceptibility and resistance concerns [8, 19]. Whether species identity, fungal burden, or host factors identify a subgroup with respiratory *Candida* spp. who might benefit from antifungal therapy remains uncertain.

Treatment analyses outside randomized trials are difficult, however, because antifungal initiation is vulnerable to immortal time bias and confounding by indication [20, 21]. Prior randomized trials and observational analyses have not shown consistent benefit from empiric antifungal therapy in critically ill patients with *Candida* spp. colonization [22]. We therefore studied hospitalized adults with respiratory *Candida* spp. without candidemia during the same admission to evaluate species distribution, clinical correlates, mortality, and the association between systemic antifungal therapy initiated after culture and mortality, using time-dependent and weighted analyses.

## METHODS

### Study Design and Setting

We performed a single-center retrospective cohort study within the Montefiore Health System, an urban safety-net system in the Bronx, New York, comprising 3 hospitals with ∼2000 beds and 84 ICU beds. Adults with respiratory *Candida* spp. were identified from 1 January 2018 through 31 December 2023.

**Figure 1.**
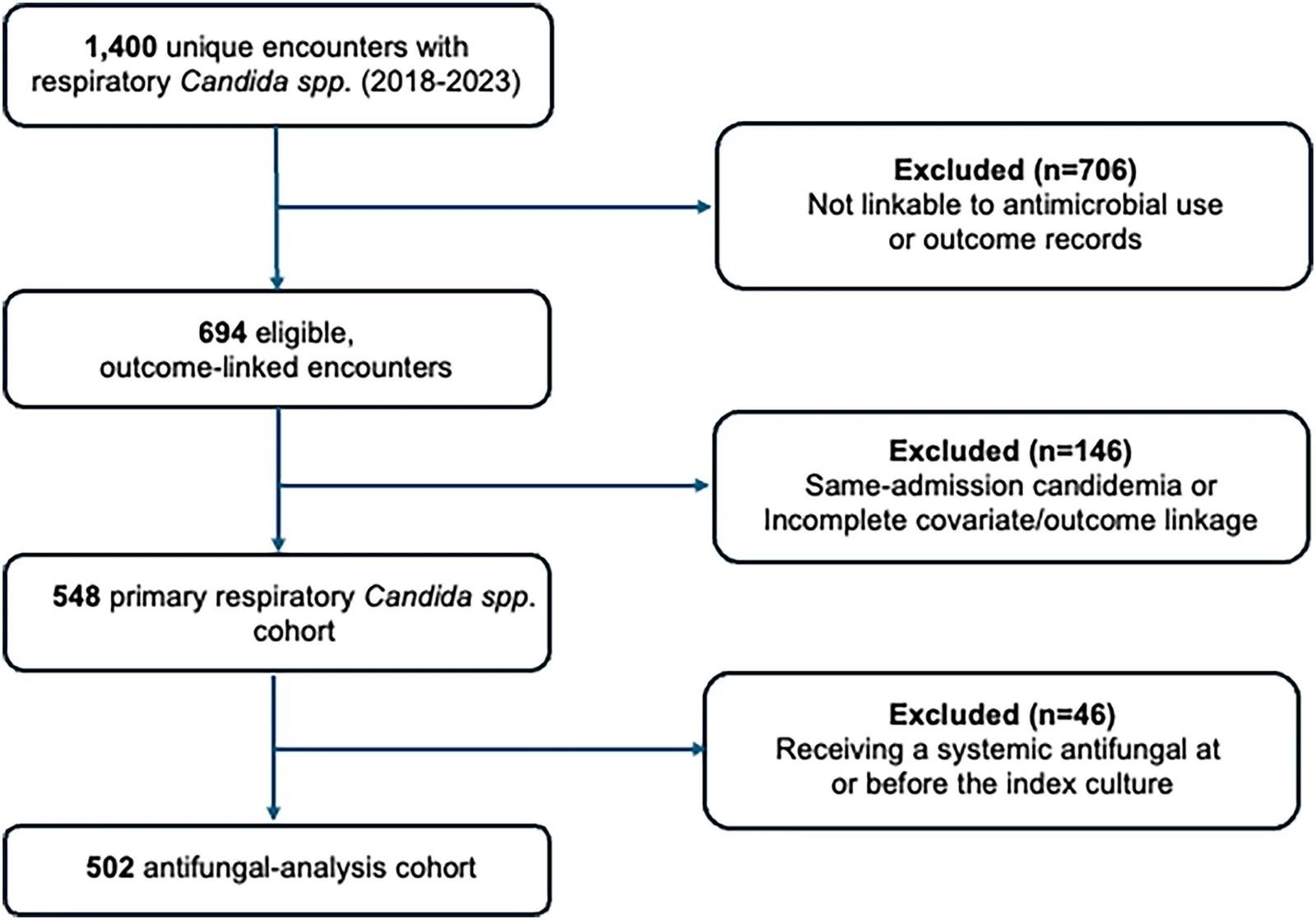
Cohort flow diagram. Of 1400 unique respiratory Candida spp. encounters (2018–2023), 694 had complete antimicrobial days-of-therapy and outcome linkage; 146 were excluded for same-admission candidemia or incomplete covariate or outcome data, leaving the primary respiratory Candida spp. cohort (N = 548). Excluding 46 patients already receiving a systemic antifungal at the index culture yielded the antifungal-analysis cohort (N = 502).

Adults aged 19 years or older were eligible. Patients were identified from the microbiology database if *Candida* spp. were isolated from an expectorated sputum, endotracheal/tracheal aspirate, or bronchoalveolar lavage specimen during hospitalization. For patients with multiple positive respiratory cultures, the first positive culture was the index culture; persistence was evaluated separately. To focus on respiratory *Candida* spp. without concurrent bloodstream infection, we excluded patients with candidemia during the same admission. A contemporaneous candidemia cohort from the same health system was retained for comparison. Figure 1 shows cohort flow.

#### Exposures and Covariates

The primary exposure was respiratory *Candida* species group, categorized as *C albicans*, *C glabrata*, or other non-*albicans Candida* spp. The secondary exposure was systemic antifungal therapy started after the index respiratory culture. Antifungal start dates and days of therapy were extracted for azoles, echinocandins, and polyenes; therapy already in use at the index culture was distinguished from therapy started afterward.

Respiratory cultures were obtained during routine care and reported qualitatively or semi-quantitatively; quantitative respiratory cultures were not routinely performed. High respiratory fungal burden was defined by moderate/many or 3+/4+ growth vs rare/few or 1+/2+ growth. Persistent respiratory recovery was defined as 2 or more *Candida*-positive respiratory culture collection events from the same encounter, requiring distinct collection times. Respiratory cultures from all 3 hospitals were processed centrally in the microbiology laboratory at the main campus using the same laboratory procedures. Because respiratory isolates were not routinely tested for antifungal susceptibility, susceptibility was summarized only for bloodstream *Candida* isolates obtained during the same period.

Microbiology and electronic health record linkage provided demographics, race and ethnicity, comorbidities, laboratory values, specimen source, bacterial co-isolation, organ support, ICU admission, and admission and discharge dates. Comorbidities were identified from International Classification of Diseases, Tenth Revision, Clinical Modification codes in the year before admission, including diabetes mellitus (E08–E13), human immunodeficiency virus (B20 or Z21), kidney, heart, lung, heart-lung, or liver transplant status (Z94.0–Z94.4), hematopoietic cell transplant (Z94.81 or Z94.84), end-stage renal disease (N18.6), and liver disease (K70–K77). End-stage renal disease was selected because it is more specifically identified using administrative diagnosis codes than chronic kidney disease. ICU admission or stay was identified from bed-transfer data. Mechanical ventilation and vasopressors were treated as markers of acute organ support.

White blood cell count, absolute neutrophil count, and absolute lymphocyte count were summarized across a 7-day window around the index culture. Lymphopenia was defined as an absolute lymphocyte count <1.0 × 10^9^/L. Serum (1,3)-β-D-glucan and C-reactive protein values were extracted when obtained as part of clinical care.

Medication data were extracted from inpatient medication administration and pharmacy records. These data included systemic antifungals, antibacterial classes mapped to National Healthcare Safety Network categories, vasopressors, and systemic corticosteroids, with start dates and days of therapy during the index hospitalization.

Vital status was ascertained from internal electronic health record date-of-death fields and external sources, including the National Death Index for patients with cancer or HIV and the institutional Cancer Registry.

#### Outcomes

Primary and secondary outcomes were all-cause death through 30 and 90 days after the index respiratory culture. The 30-day outcome captured mortality near the index culture and treatment decision, whereas the 90-day outcome captured later mortality, including after discharge. Both in-hospital and post-discharge deaths were included. Day 0 was the index respiratory culture date. Patients without documented death were censored at the last available vital-status date.

#### Statistical Analysis

Continuous variables were summarized as medians with interquartile ranges and compared with Wilcoxon rank-sum or Kruskal-Wallis tests. Categorical variables were compared with chi-square or Fisher exact tests. Kaplan-Meier estimates and log-rank tests compared unadjusted survival. Correlates of non-albicans *Candida* species and antifungal receipt were evaluated with multivariable logistic regression. Associations with death through 30 and 90 days were estimated with multivariable Cox proportional hazards models; proportional hazards assumptions were assessed with Schoenfeld residuals. Primary mortality models adjusted for species group, age, sex, race and ethnicity, immune-status category, end-stage renal disease, liver disease, mechanical ventilation, vasopressor use, and high respiratory fungal burden.

Antifungal therapy was evaluated among patients not receiving systemic antifungals at the index culture. Starting antifungal therapy after the index culture was the exposure in the primary model and was modeled as time-dependent, with patients contributing unexposed person-time before initiation and exposed person-time afterward. This approach was used to reduce immortal time bias [20, 21, 23]. The model adjusted for age, sex, species group, vasopressor use, and high respiratory fungal burden. Stabilized inverse-probability-of-treatment weights trimmed at the first and 99th percentiles addressed measured confounding in a sensitivity analysis. Sensitivity analyses included a 7-day landmark analysis, restriction to high-burden cultures, immune status and persistence analyses, a COVID-19-era analysis, and a time-fixed azole analysis. For landmark analyses, patients who died before day 7 were excluded, antifungal exposure and persistence were classified using data through day 7, and follow-up began at the day-7 landmark. Analyses used Stata/SE 16.1 (StataCorp LLC, College Station, Texas). Two-sided *P* < .05 was considered statistically significant. *P*-values were interpreted descriptively without adjustment for multiple comparisons.

#### Patient Consent Statement

The Albert Einstein College of Medicine Institutional Review Board approved the study with a waiver of informed consent (IRB number: 2024-15952).

## RESULTS

### Cohort Characteristics and Species Distribution

After antimicrobial and outcome linkage, 694 respiratory *Candida* spp. encounters were eligible. We excluded 146 because of candidemia during the same admission or incomplete covariate or outcome data, leaving 548 patients (Table 1; Figure 1). Median age was 66 years (IQR, 57–75); 301 (54.9%) were male, 183 (33.4%) were non-Hispanic Black, and 184 (33.6%) were Hispanic. Diabetes mellitus was present in 230 patients (42.0%), end-stage renal disease in 37 (6.8%), and solid organ transplant in 41 (7.5%; kidney, 26; lung, 11; heart, 3; liver, 1). Patients were critically ill: 458 (83.6%) required ICU admission, 429 (78.3%) required mechanical ventilation, and 396 (72.3%) received vasopressors. Broad-spectrum antibiotic exposure was common, including glycopeptides in 494 patients (90.1%), β-lactams in 449 (81.9%), and carbapenems in 229 (41.8%).

**Table 1.** Clinical Characteristics by Candida spp. Group (Primary Respiratory Cohort, N = 548)

Characteristic	Overall (N = 548)	*C albicans* (N = 478)	*C glabrata* (N = 18)	Other Non-albicans (N = 52)	*P-*value
Demographics					
Age, years, median (IQR)	66 (57–75)	67 (57–76)	67 (54–76)	63 (57–72)	.24
Male sex	301 (54.9)	258 (54.0)	11 (61.1)	32 (61.5)	.50
Non-Hispanic Black	183 (33.4)	158 (33.1)	10 (55.6)	15 (28.8)	.11
Hispanic	184 (33.6)	155 (32.4)	5 (27.8)	24 (46.2)	.12
Non-Hispanic White	86 (15.7)	80 (16.7)	1 (5.6)	5 (9.6)	.20
Other/Unknown/Asian	95 (17.3)	85 (17.8)	2 (11.1)	8 (15.4)	.71
Body mass index, median (IQR)	27 (22–32)	27 (22–32)	26 (23–31)	26 (23–30)	.68
Immune status					
Non-immunocompromised	444 (81.0)	401 (83.9)	7 (38.9)	36 (69.2)	<.001
HIV	56 (10.2)	42 (8.8)	7 (38.9)	7 (13.5)	<.001
Solid organ transplant	41 (7.5)	29 (6.1)	3 (16.7)	9 (17.3)	.004
Hematopoietic cell transplant	7 (1.3)	6 (1.3)	1 (5.6)	0 (0.0)	.19
Comorbidities					
Diabetes mellitus	230 (42.0)	198 (41.4)	8 (44.4)	24 (46.2)	.79
End-stage renal disease	37 (6.8)	27 (5.6)	2 (11.1)	8 (15.4)	.02
Liver disease	38 (6.9)	29 (6.1)	3 (16.7)	6 (11.5)	.09
Acute severity and exposures					
ICU admission	458 (83.6)	398 (83.3)	16 (88.9)	44 (84.6)	.80
Mechanical ventilation	429 (78.3)	378 (79.1)	16 (88.9)	35 (67.3)	.08
Vasopressor use	396 (72.3)	348 (72.8)	13 (72.2)	35 (67.3)	.70
Corticosteroid use	302 (55.1)	259 (54.2)	13 (72.2)	30 (57.7)	.30
High fungal burden	382 (69.7)	337 (70.5)	11 (61.1)	34 (65.4)	.54
Funguria (concurrent)	91 (16.6)	86 (18.0)	0 (0.0)	5 (9.6)	.05
Antibiotic exposure during hospitalization					
Glycopeptides (vancomycin)	494 (90.1)	433 (90.6)	17 (94.4)	44 (84.6)	.32
β-Lactams	449 (81.9)	394 (82.4)	15 (83.3)	40 (76.9)	.61
Cephalosporins	428 (78.1)	372 (77.8)	16 (88.9)	40 (76.9)	.53
Carbapenems	229 (41.8)	188 (39.3)	11 (61.1)	30 (57.7)	.009
Fluoroquinolones	115 (21.0)	97 (20.3)	4 (22.2)	14 (26.9)	.53
Respiratory specimen source					
Expectorated sputum	198 (36.1)	173 (36.2)	5 (27.8)	20 (38.5)	.72
Tracheal aspirate	297 (54.2)	270 (56.5)	10 (55.6)	17 (32.7)	.005
Bronchoalveolar lavage	53 (9.7)	35 (7.3)	3 (16.7)	15 (28.8)	<.001
Respiratory bacterial co-isolation					
Any bacterial co-isolate	84 (15.3)	76 (15.9)	0 (0.0)	8 (15.4)	.19
*Staphylococcus aureus* (16 MRSA, 11 MSSA)	27 (4.9)	26 (5.4)	0 (0.0)	1 (1.9)	.33
Host and inflammatory markers					
WBC, ×10^9^/L, median (IQR)	11.9 (8.9–16.8)	11.9 (9.0–16.9)	9.0 (5.1–13.7)	12.2 (8.8–15.7)	.16
ANC, ×10^9^/L, median (IQR)	9.3 (6.8–13.7)	9.3 (6.8–13.8)	5.5 (2.7–10.4)	9.8 (7.0–13.6)	.03
ALC, ×10^9^/L, median (IQR)	1.05 (0.70–1.53)	1.06 (0.71–1.52)	0.84 (0.59–1.57)	0.98 (0.68–1.59)	.66
Lymphopenia (ALC <1.0)	247 (45.2)	210 (44.0)	11 (61.1)	26 (50.0)	.27
Antifungal therapy					
Any systemic antifungal	148 (27.0)	117 (24.5)	10 (55.6)	21 (40.4)	.001
Azole	111 (20.3)	88 (18.4)	6 (33.3)	17 (32.7)	.02
Echinocandin	98 (17.9)	80 (16.7)	5 (27.8)	13 (25.0)	.18
Polyene	20 (3.6)	15 (3.1)	2 (11.1)	3 (5.8)	.14
Outcomes					
30-day mortality	148 (27.0)	132 (27.6)	9 (50.0)	7 (13.5)	.008
90-day mortality, %	46.0	47.2	62.1	29.2	.02

Data are n (%) unless otherwise indicated. *P*-values compare species groups (Kruskal-Wallis for continuous variables; chi-square or Fisher exact test for categorical variables). Other non-albicans includes *C dubliniensis*, *C parapsilosis*, *C tropicalis*, *C lusitaniae*, *C krusei*, *C auris*, and rare *Candida* spp.

Abbreviations: ALC, absolute lymphocyte count; ANC, absolute neutrophil count; ICU, intensive care unit; IQR, interquartile range; MRSA, methicillin-resistant *Staphylococcus aureus*; MSSA, methicillin-susceptible *Staphylococcus aureus*; WBC, white blood cell count.

*C albicans* accounted for 478 isolates (87.2%), *C glabrata* for 18 (3.3%), and other non-albicans *Candida* spp. for 52 (9.5%; *C dubliniensis*, 17; *C parapsilosis*, 12; *C tropicalis*, 11; *C lusitaniae*, 5; *C krusei*, 2; *C auris*, 1; rare species, 4). From 2019 to 2023, non-albicans *Candida* spp. increased from 7.2% to 18.3% in respiratory cultures (Supplementary Figure 1). In contrast, non-albicans species were already predominant and relatively stable in blood cultures from the contemporaneous candidemia cohort (Supplementary Figure 1).

Bacterial respiratory co-isolation occurred in 84 patients (15.3%): 76 with *C albicans*, none with *C glabrata*, and 8 with other non-*albicans Candida* spp. (Table S1). *Staphylococcus aureus* was the most common co-isolate (n = 27; 16 methicillin-resistant and 11 methicillin-susceptible), followed by *Klebsiella* spp. (n = 23) and *Pseudomonas aeruginosa* (n = 15).

### Correlates of Non-albicans Candida spp.

In multivariable logistic regression, non-albicans *Candida* spp. were associated with HIV (adjusted odds ratio [aOR], 2.73; 95% confidence interval [CI], 1.29–5.76), solid organ transplant (aOR, 3.02; 95% CI, 1.21–7.57), and carbapenem use (aOR, 2.57; 95% CI, 1.49–4.44) (Table S2). End-stage renal disease had a higher, nonsignificant odds ratio.

### Mortality and Predictors

**Figure 2.**
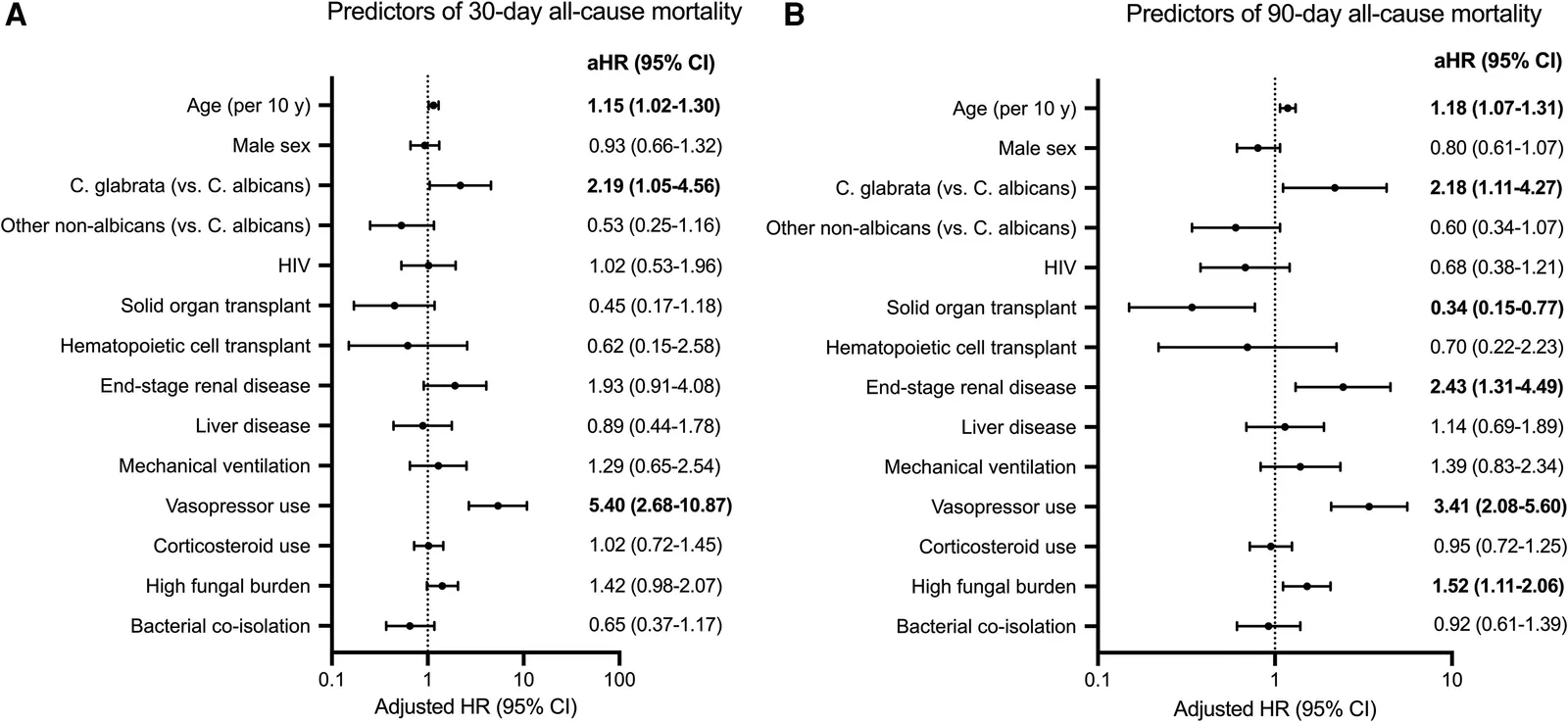
Predictors of all-cause mortality (multivariable Cox models). *A*, 30-day mortality (N = 548). *B*, 90-day mortality (N = 548, with incomplete follow-up censored at last contact). Circles show adjusted hazard ratios and bars the 95% CI; the vertical line marks hazard ratio = 1. Species groups are referenced to *C albicans*; each panel shows every covariate included in the model.

Thirty-day mortality was 27.0% (148/548) and differed by species group: 27.6% with *C albicans*, 50.0% with *C glabrata*, and 13.5% with other non-albicans *Candida* spp. (*P* = .008) (Figure 2A). After adjustment, *C glabrata* was associated with higher 30-day mortality than *C albicans* (adjusted hazard ratio [aHR], 2.19; 95% CI, 1.05–4.56), whereas other non-albicans *Candida* spp. were not (aHR, 0.53; 95% CI, 0.25–1.16). Vasopressor use (aHR, 5.40; 95% CI, 2.68–10.87) and age per 10-year increase (aHR, 1.15; 95% CI, 1.02–1.30) were also associated with 30-day mortality.

Ninety-day mortality was 46.0% and differed by species group: 47.2% with *C albicans*, 62.1% with *C glabrata*, and 29.2% with other non-albicans *Candida* spp. (*P* = .02) (Figure 2B). *C glabrata* remained associated with higher 90-day mortality (aHR, 2.18; 95% CI, 1.11–4.27), whereas other non-albicans *Candida* spp. were not (aHR, 0.60; 95% CI, 0.34–1.07). Vasopressor use (aHR, 3.41; 95% CI, 2.08–5.60), age per 10-year increase (aHR, 1.18; 95% CI, 1.07–1.31), high fungal burden (aHR, 1.52; 95% CI, 1.11–2.06), and end-stage renal disease (aHR, 2.43; 95% CI, 1.31–4.49) were also associated with higher 90-day mortality. Solid organ transplant was associated with lower 90-day mortality (aHR, 0.34; 95% CI, 0.15–0.77).

The *C glabrata* association persisted in several sensitivity analyses (Supplementary Figure 2), including the 7-day landmark analysis (aHR, 3.37; 95% CI, 1.30–8.71), restriction to high-burden cultures (aHR, 3.46; 95% CI, 1.48–8.05), and the immunocompromised stratum (HIV, solid organ transplant, or hematopoietic cell transplant; n = 104; aHR, 4.88; 95% CI, 1.40–16.95). Proportional hazards assumptions were not violated in the primary 30-day model (Schoenfeld *P* = .94). In exploratory analyses, *C glabrata* mortality was high in lower-airway samples (70% [7/10] in tracheal aspirates) and expectorated sputum (40% [2/5]). *C glabrata* was more often persistent than *C albicans* (2 or more positive respiratory cultures by day 7, 50.0% vs 24.0%). In the 7-day landmark analysis, the risk was concentrated among patients with persistent respiratory recovery (aHR, 4.66; 95% CI, 1.47–14.73) and absent after a single positive respiratory culture (aHR, 0.79; 95% CI, 0.17–3.63).

The association attenuated when COVID-19 surge years (2020–2021) were excluded (aHR, 1.57; 95% CI, 0.52–4.72) and was stronger during 2020–2021 (aHR, 3.72; 95% CI, 1.02–13.59). Remdesivir, used as a proxy for COVID-19, was recorded in 34 patients (6.2%) and peaked at 20.0% in 2021; 31 of 34 (91.2%) also received corticosteroids. Adjustment for remdesivir attenuated but did not reverse the *C glabrata* estimate. *Aspergillus* spp. were recovered from 5 respiratory cultures (0.9%), all with *C albicans* and none among remdesivir-exposed patients.

### Host and Inflammatory Markers

Patients with *C glabrata* had lower neutrophil counts than patients with other species (median absolute neutrophil count, 5.5 vs 9.3 × 10^9^/L; *P* = .03), and lymphopenia was common overall (45.2%) and among patients with *C glabrata* (61.1%) (Table 1). Lymphopenia was associated with 30-day mortality after adjustment (aHR, 1.74; 95% CI, 1.24–2.43). Serum (1,3)-β-D-glucan was measured in 111 patients (20.3%); 44.1% were positive (≥80 pg/mL), but positivity was not associated with 30-day mortality. C-reactive protein was measured in 77 patients (14.1%); higher values were associated with 30-day mortality (aHR per 10 mg/L, 1.21; 95% CI, 1.01–1.46).

### Antifungal Treatment and Mortality

**Figure 3.**
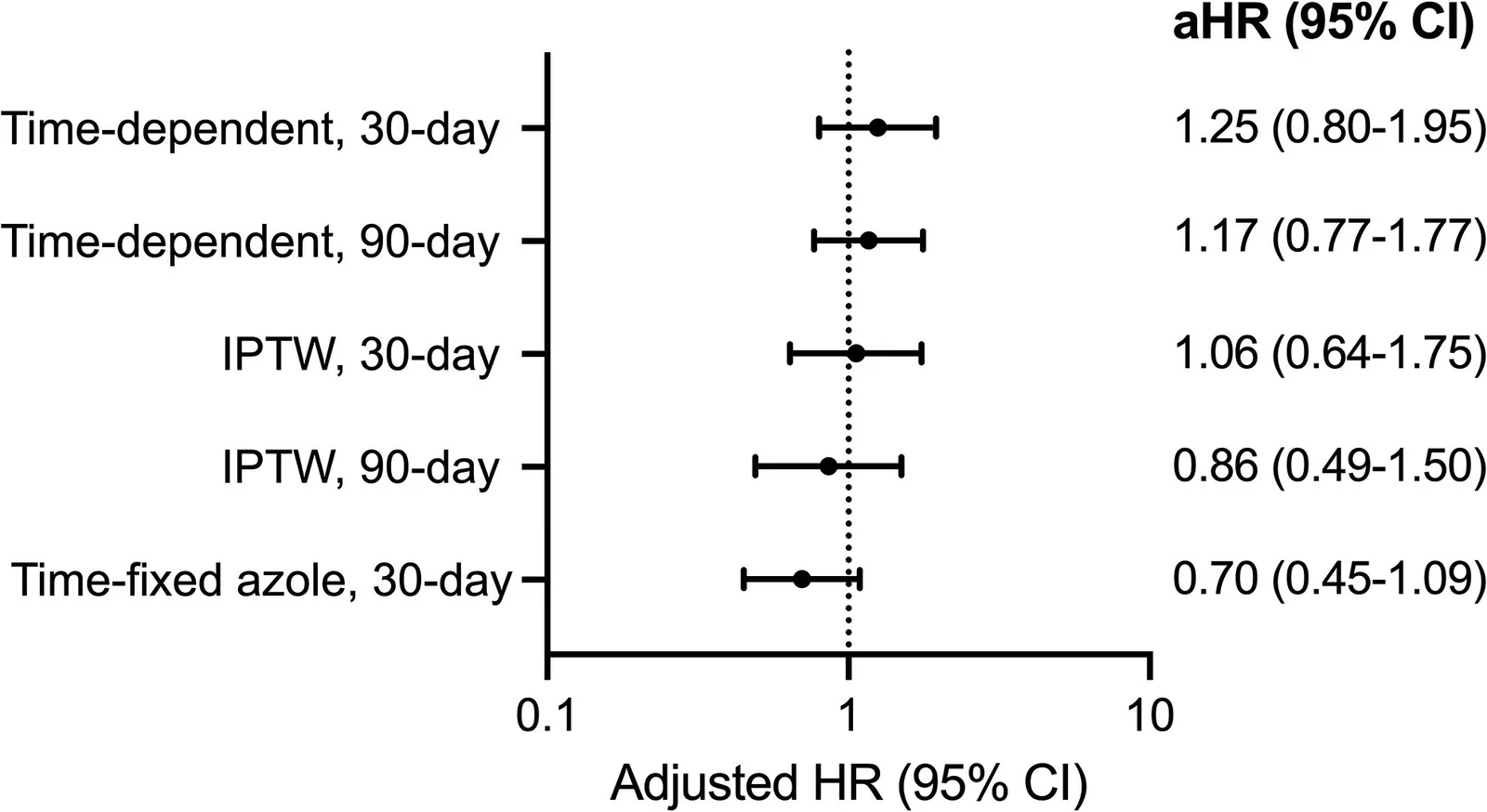
Antifungal therapy and mortality among patients not already receiving a systemic antifungal at the index culture (N = 502). Circles show hazard ratios and bars the 95% CI; the vertical line marks hazard ratio = 1. Estimates are shown for time-dependent and stabilized inverse-probability-of-treatment-weighted (IPTW) models at 30 and 90 days and for a conventional time-fixed azole model; values >1 indicate higher mortality with antifungal therapy.

Any systemic antifungal was received by 148 patients (27.0%), including 117 (24.5%) with *C albicans*, 10 (55.6%) with *C glabrata*, and 21 (40.4%) with other non-albicans *Candida* spp. (*P* = .001) (Table 1). Class-level exposures included azoles in 111 patients (20.3%), echinocandins in 98 (17.9%), and polyenes in 20 (3.6%). Forty-six patients (8.4%) were already receiving antifungals at the index culture, and 102 (18.6%) initiated therapy afterward at a median of 4.4 days after collection of the respiratory specimen. Median duration was 8 days (IQR, 4–16) (Figure 3).

Patients who received antifungals differed from untreated patients (Supplementary Table 3). In multivariable models, antifungal receipt was more likely with *C glabrata* (aOR, 3.74; 95% CI, 1.32–10.60), other non-albicans *Candida* spp. (aOR, 2.11; 95% CI, 1.11–4.02), solid organ transplant (aOR, 3.80; 95% CI, 1.62–8.94), and hematopoietic cell transplant (aOR, 6.77; 95% CI, 1.22–37.65). Receipt was less likely with older age (aOR per 10 years, 0.81; 95% CI, 0.71–0.93) and among non-Hispanic Black patients (aOR, 0.57; 95% CI, 0.34–0.94) and Hispanic patients (aOR, 0.61; 95% CI, 0.37–1.00).

Among 502 patients not already receiving a systemic antifungal at the index culture, time-dependent antifungal therapy was not associated with lower 30-day mortality (aHR, 1.25; 95% CI, 0.80–1.95) or 90-day mortality (aHR, 1.17; 95% CI, 0.77–1.77). Stabilized inverse probability weighted analyses were also null at 30 days (HR, 1.06; 95% CI, 0.64–1.75) and 90 days (HR, 0.86; 95% CI, 0.49–1.50) (Figure 3). A time-fixed azole model gave a more favorable but nonsignificant estimate (aHR, 0.70; 95% CI, 0.45–1.09).

### Comparison With Candidemia

The contemporaneous candidemia cohort comprised 379 patients. Overall 90-day mortality was similar (47.5% in candidemia vs 46.0% in the respiratory cohort), but species distribution differed. *C glabrata* accounted for 122 candidemia cases (32.2%) compared with 18 respiratory cases (3.3%) (Supplementary Table 4; Supplementary Figure 1). In candidemia, *C glabrata* was not associated with higher 90-day mortality than *C albicans* (age-adjusted HR, 0.91; 95% CI, 0.64–1.31). Candidemia susceptibility data showed that *C glabrata* was predominantly fluconazole susceptible-dose-dependent, with 5.5% fluconazole resistance and 2.0% micafungin resistance, whereas *C albicans* was 98.9% fluconazole susceptible (Supplementary Table 5).

## DISCUSSION

In this diverse, single-center cohort of critically ill hospitalized adults with respiratory *Candida* spp. and extensive antibiotic exposure, respiratory recovery identified a high-risk population but not a group that appeared to benefit from antifungal therapy. Ninety-day mortality was 46.0%, similar to the contemporaneous candidemia cohort. *C glabrata* was uncommon but associated with higher 30- and 90-day mortality, particularly with high-burden or persistent recovery. Systemic antifungal initiation was not associated with lower mortality after accounting for treatment timing and measured confounding. These findings suggest that respiratory *Candida* spp., especially *C glabrata*, may be more informative about host state and airway context than a treatable *Candida*-attributable disease process.

Our findings add species-level data to prior North American and European cohorts. In the French OUTCOMEREA cohort, respiratory *Candida* spp. colonization was associated with longer ICU and hospital stays and subsequent *Pseudomonas* ventilator-associated pneumonia, but not mortality [13]. *C glabrata* accounted for 20.1% of isolates. Respiratory *Candida* spp. have also been linked to subsequent *Acinetobacter baumannii* ventilator-associated pneumonia in clinical and experimental studies [24, 25]. In contrast, the prospective FUNGIBACT cohort found no association between bronchial colonization with *Candida* spp. and bacterial ventilator-associated pneumonia overall after adjustment for time-dependent confounders and immune function [26]. These data support respiratory *Candida* spp. as a marker of a vulnerable host and altered airway environment, not a direct cause of bacterial pneumonia.

European autopsy data showed that positive respiratory cultures poorly identify invasive *Candida* pneumonia [10]. Tissue diagnosis is rarely available, and clinicians are often left to interpret culture positivity in a critically ill patient without a reliable biomarker of disease attribution. In most patients, colonization remains the appropriate interpretation. In selected hosts, however, respiratory recovery may inform illness pathophysiology and expected course. Prior studies show that organisms dismissed as normal respiratory flora can cause pneumonia in susceptible hosts [27], and *Candida* spp. may contribute to disease in chronic aspiration when high-quality sputum shows abundant intracellular yeast or pseudohyphae, high quantitative burden, and bacterial co-isolation [28]. Our cohort lacked histopathology, quantitative cultures, and adjudicated pneumonia diagnoses needed to establish *Candida*-attributable pneumonia. Yet, the association of *C glabrata* identity, burden, and persistence with mortality supports a related host-microbial concept that respiratory *Candida* spp. may carry clinically useful information, even when culture positivity alone is not a treatment indication.

Species identity likely reflects both microbial selection and host state. Experimental studies show that *C albicans* can form polymicrobial biofilms, enhance bacterial virulence, and impair alveolar macrophage function, potentially favoring bacterial coinfection [15–18]. In our study, non-albicans *Candida* spp. were associated with HIV, solid organ transplant, and carbapenem exposure, implicating host vulnerability and antimicrobial pressure. No bacterial co-isolates were detected in the small *C glabrata* subgroup despite higher carbapenem exposure. Lower neutrophil counts, lymphopenia, persistent recovery, and attenuation of the association outside 2020–2021 further suggest that respiratory *C glabrata* may be a sentinel of host vulnerability and disrupted airway microbiology. The 2020–2021 pandemic period may have made this signal more apparent through differences in patient population, corticosteroid exposure, antimicrobial pressure, and airway microbiology.

Our antifungal findings reinforce the cautious stance reflected in current guidelines [8, 9]. Empiric anidulafungin did not improve clinical outcomes in patients with suspected ventilator-associated pneumonia and *Candida*-positive airway secretions (CANTREAT, NCT00934934) [29]. Empiric fluconazole did not improve outcomes in high-risk ICU patients [30]. Caspofungin prophylaxis did not reduce invasive candidiasis in high-risk critically ill adults (MSG-01, NCT00520234) [31]. Empiric micafungin did not improve day-28 survival free of invasive fungal infection in ICU-acquired sepsis with *Candida* spp. colonization (EMPIRICUS, NCT01773876) [32]. A 2024 meta-analysis found no clinical benefit from antifungal therapy in mechanically ventilated patients with pulmonary Candida colonization [33], and a marginal structural model analysis found no survival benefit from empirical systemic antifungals in ventilated critically ill adults [34]. Our analysis likewise did not identify improved survival when treatment timing was modeled. The more favorable time-fixed azole estimate likely reflects immortal time and treatment-selection bias [20, 21]. A null overall estimate does not exclude a small subgroup that may benefit, but we did not identify one. Therapy is often started after critical illness is established, which may limit its ability to modify outcomes if an occult fungal disease process is already advanced.

Solid organ transplant was associated with lower 90-day mortality, consistent with sepsis studies in which transplant recipients had similar or better adjusted survival than nontransplant patients [35, 36]. Transplant recipients were also more likely to receive antifungals, and this imbalance may have contributed to the more favorable time-fixed azole estimate. Antifungal receipt was lower among non-Hispanic Black and Hispanic patients after adjustment. Because treatment indication, infectious diseases consultation, and time-updated severity data were unavailable, we could not determine whether these differences reflected appropriate clinical decision-making, residual confounding by indication, or potential differences in care delivery.

### Strengths and Limitations

Strengths include a real-world cohort from an urban safety-net system serving many Black and Hispanic patients, populations underrepresented in fungal infection research [37, 38]. We linked microbiology, antimicrobial days of therapy, laboratory, organ support, and outcome data and used time-dependent, landmark, and weighted analyses to reduce immortal time bias and confounding by indication.

Limitations include the retrospective single-center design, residual confounding, the absence of a contemporaneous respiratory *Candida*-negative control, and nonrandom antifungal exposure. We lacked Sequential Organ Failure Assessment and Acute Physiology and Chronic Health Evaluation II scores, treatment indications, infectious diseases consultation, time-updated severity, quantitative cultures, histopathology, specimen collection and quality measures, respiratory isolate susceptibility data, and adjudicated causes of death. We therefore could not estimate *Candida*- or ventilator-associated pneumonia-attributable mortality. Antifungal exposure was captured as class-level start dates and days of therapy, without enough detail to judge agent selection. We lacked quantitative measures of fungal clearance and evaluated treatment effect using mortality alone. In this critically ill population, mortality is influenced by many measured and unmeasured factors and may not be the best outcome measure to detect an antifungal effect. We therefore could not determine whether therapy improved clearance or provided clinical benefit, particularly in patients with high fungal burden.

COVID-19 overlapped the study period and was not directly coded; remdesivir was an imperfect proxy. Most *C glabrata* isolates and deaths occurred during 2020–2021, and the association attenuated outside those years. Pandemic-related changes in ICU case mix, corticosteroid exposure, antimicrobial pressure, and microbial epidemiology therefore remain possible explanations. The *C glabrata* subgroup was small (n = 18), and adjusted estimates may be affected by sparse-data bias.

## CONCLUSION

Respiratory *Candida* spp. identified a critically ill, high-risk population. In analyses accounting for treatment timing and measured confounding, systemic antifungal therapy was not associated with improved survival. *C glabrata* identified a small, high-mortality subgroup, likely reflecting vulnerable host state and airway microbiology disruption from antimicrobial and other iatrogenic exposures. Species identity, fungal burden, persistence, and local epidemiology may inform interpretation, but respiratory culture positivity alone may not provide sufficient evidence for antifungal therapy without other evidence of invasive candidiasis or another treatable fungal syndrome. Identifying any subgroup that benefits will require validated markers that distinguish high-risk colonization from *Candida*-attributable disease.

## Supplementary Material

ofag550_Supplementary_Data

## Supplementary Data

Supplementary materials are available at *Open Forum Infectious Diseases* online. Consisting of data provided by the authors to benefit the reader, the posted materials are not copyedited and are the sole responsibility of the authors, so questions or comments should be addressed to the corresponding author.
