# Supplementary material for: No Survival Benefit Associated With Antifungal Therapy for Respiratory Candida spp.: a Single-Center Retrospective Cohort Study
Source: Open Forum Infect Dis. 2026 Sep 3;13(9):ofag550. doi: 10.1093/ofid/ofag550 (PMC13579478; doi:10.1093/ofid/ofag550)
Supplement: Supplementary file 1 [file ofag550_supplementary_data.docx]

**Supplementary Tables S1-S5 and Supplementary Figures S1-S2**

**Table S1. Respiratory bacterial co-isolates by *Candida* spp. group (N = 548).**

| **Bacterial co-isolate** | **Overall (N=548)** | ***C. albicans* (478)** | ***C. glabrata* (18)** | **Other (52)** |
| --- | --- | --- | --- | --- |
| *Staphylococcus aureus* (16 MRSA, 11 MSSA) | 27 (4.9) | 26 (5.4) | 0 (0.0) | 1 (1.9) |
| *Klebsiella* spp. | 23 (4.2) | 21 (4.4) | 0 (0.0) | 2 (3.8) |
| *Pseudomonas aeruginosa* | 15 (2.7) | 13 (2.7) | 0 (0.0) | 2 (3.8) |
| *Enterobacter* spp. | 11 (2.0) | 10 (2.1) | 0 (0.0) | 1 (1.9) |
| *Escherichia coli* | 9 (1.6) | 8 (1.7) | 0 (0.0) | 1 (1.9) |
| *Enterococcus* spp. | 5 (0.9) | 5 (1.0) | 0 (0.0) | 0 (0.0) |
| *Serratia* spp. | 2 (0.4) | 1 (0.2) | 0 (0.0) | 1 (1.9) |
| *Acinetobacter* spp. | 2 (0.4) | 2 (0.4) | 0 (0.0) | 0 (0.0) |
| *Stenotrophomonas maltophilia* | 1 (0.2) | 1 (0.2) | 0 (0.0) | 0 (0.0) |
| *Streptococcus pneumoniae* | 1 (0.2) | 1 (0.2) | 0 (0.0) | 0 (0.0) |
| **Any bacterial co-isolate** | **84 (15.3)** | **76 (15.9)** | **0 (0.0)** | **8 (15.4)** |

Data are n (%) of patients with the organism co-isolated from a respiratory culture during the index hospitalization. Categories are not mutually exclusive. MRSA, *methicillin-resistant S. aureus*; MSSA, *methicillin-susceptible S. aureus*.

**Table S2. Variables associated with non-albicans *Candida* spp. (vs *C. albicans*) in multivariable logistic regression (N = 548).**

| **Variable** | **Adjusted OR (95% CI)** | ***P*-value** |
| --- | --- | --- |
| HIV | 2.73 (1.29–5.76) | .008 |
| Solid organ transplant | 3.02 (1.21–7.57) | .02 |
| End-stage renal disease | 1.70 (0.63–4.59) | .30 |
| Carbapenem use | 2.57 (1.49–4.44) | <.001 |
| Age, per 10 years | 0.94 (0.78–1.14) | .55 |
| Male sex | 1.58 (0.90–2.77) | .11 |
| Non-Hispanic Black (vs. non-Hispanic White) | 2.34 (0.88–6.23) | .09 |
| Hispanic (vs. non-Hispanic White) | 2.27 (0.86–6.02) | .10 |

**Table S3. Variables associated with systemic antifungal receipt in multivariable logistic regression (N = 548).**

| **Predictor** | **Adjusted OR (95% CI)** | ***P*-value** |
| --- | --- | --- |
| *C. glabrata* (vs *C. albicans*) | 3.74 (1.32–10.60) | .01 |
| Other non-albicans *Candida* spp. (vs *C. albicans*) | 2.11 (1.11–4.02) | .02 |
| Age, per 10 years | 0.81 (0.71–0.93) | .003 |
| HIV (vs non-immunocompromised) | 1.27 (0.62–2.61) | .52 |
| Solid organ transplant (vs non-immunocompromised) | 3.80 (1.62–8.94) | .002 |
| Hematopoietic cell transplant (vs non-immunocompromised) | 6.77 (1.22–37.65) | .03 |
| Non-Hispanic Black (vs non-Hispanic White) | 0.57 (0.34–0.94) | .03 |
| Hispanic (vs non-Hispanic White) | 0.61 (0.37–1.00) | .052 |
| High fungal burden (vs low) | 0.75 (0.49–1.16) | .20 |
| Mechanical ventilation | 1.14 (0.58–2.25) | .71 |
| Vasopressor use | 1.77 (0.95–3.30) | .07 |
| End-stage renal disease | 0.44 (0.16–1.25) | .12 |

Outcome was receipt of any systemic antifungal during the index hospitalization (148 patients, 27.0%). The model included only the variables shown. OR, odds ratio; CI, confidence interval.

**Table S4. Respiratory versus candidemia cohorts: species distribution and species-specific 90-day mortality.**

| **Characteristic** | **Respiratory cohort (N=548)** | **Candidemia cohort (N=379)** |
| --- | --- | --- |
| **Species distribution, n (%)** | | |
| *C. albicans* | 478 (87.2) | 133 (35.1) |
| *C. glabrata* | 18 (3.3) | 122 (32.2) |
| Other non-albicans | 52 (9.5) | 124 (32.7) |
| **90-day all-cause mortality** | | |
| Overall | 46.0% | 47.5% |
| *C. albicans* | 47.2% | 47.4% |
| *C. glabrata* | 62.1% | 45.1% |
| Other non-albicans | 29.2% | 50.0% |
| **C. glabrata vs C. albicans** | | |
| Hazard ratio (95% CI) | **2.18 (1.11–4.27)** | **0.91 (0.64–1.31)** |

**Table S5. Antifungal susceptibility of bloodstream *Candida* isolates, 2018-2023.**

| **Species** | **Isolates, n** | **Fluconazole S, %** | **Fluconazole SDD, %** | **Fluconazole R, %** | **Micafungin R, %** |
| --- | --- | --- | --- | --- | --- |
| *C. albicans* | 276 | 98.9 | 0.7 | 0.4 | 0.0 |
| *C. glabrata* | 237 | 0.4 | 94.1 | 5.5 | 2.0 |
| *C. parapsilosis* | 128 | 93.8 | 2.3 | 3.9 | 0.0 |
| *C. tropicalis* | 82 | 79.3 | 8.5 | 12.2 | 0.0 |

Data are the percentage of unique isolates in each category as interpreted by the clinical microbiology laboratory (CLSI methods) for isolates with an available interpretation; 1,346 unique bloodstream isolates from 772 patients. Fluconazole categories are susceptible (S), susceptible-dose-dependent (SDD), and resistant (R).

**Supplementary Figures**

**Figure S1. Candida species composition by year, 2019-2023.**

(A) Respiratory *Candida* spp. composition by year

(B) Candidemia composition by year

Bars show the percentage of patients whose index isolate was *C. albicans*, *C. glabrata*, or other non-albicans *Candida* spp. (A) Primary respiratory cohort (N = 548), by index respiratory culture year; non-albicans species rose from 7.2% in 2019 to 18.3% in 2023. (B) Contemporaneous candidemia cohort by index blood culture year (N = 376 from 2019-2023); non-albicans species predominated and remained relatively stable across the period.

**Figure S2. Sensitivity and subgroup analyses of the *C. glabrata* 30-day mortality association (vs *C. albicans*).**

Circles show adjusted hazard ratios and bars the 95% CI; the vertical line marks hazard ratio = 1. Estimates are shown for the primary model, a 7-day landmark analysis, restriction to high-burden cultures, persistent recovery (two or more positive cultures by day 7), and both exclusion of and restriction to the 2020-2021 COVID-19 surge years.
